# When the Stars Misfire: Astrocytic Dysfunctions in Major Depressive Disorder

**DOI:** 10.1007/s11064-025-04483-y

**Published:** 2025-07-12

**Authors:** Candela González-Arias, Gertrudis Perea

**Affiliations:** Cajal Institute, CSIC, Av. Doctor Arce, 37, Madrid, 28002 Spain

## Abstract

Major depressive disorder (MDD) is a global public health concern and the leading cause of disability worldwide. It severely impairs cognitive, emotional, and social functioning, and is associated with attention, memory, and executive function deficits. MDD was traditionally considered as a neuropsychiatric disorder focused on neuronal dysfunction; however, growing evidence recognizes glial cells, particularly astrocytes, as crucial cells for the pathophysiology and pathogenesis of MDD. Astrocytes play essential roles in the central nervous system, including neurotransmitter uptake, metabolic support, neurotrophic factor production, and synaptic regulation. Evidence from MDD human patients, as well as studies involving animal models have revealed the astrocytic dysfunction as a critical factor in the pathophysiology of MDD, pointing to widespread alterations in astrocyte density, morphology, and function. This review summarizes current knowledge regarding astrocytes and MDD and discuss the potential of targeting astrocytes as a novel avenue for antidepressant development.

## Introduction

Major depressive disorder (MDD) is a chronic, heterogeneous and severe mood disorder that afflicts up to 20% of the global population, profoundly affects an individual’s quality of life and is the leading cause of disability worldwide [1, 2]. This psychiatric condition is characterized by cognitive and psychosocial impairments, leading to reduced social interaction and altered emotional information processing [3]. These deficits are accompanied by disruptions in attention, executive functions, learning, and memory processes [4].

Moreover, MDD is a recurrent, and potentially life-threatening mental disorder [5], with a considerable socioeconomic impact [6], due to its high prevalence, its onset during active periods of adulthood, and the slow-acting and limited efficacy of available treatments [7, 8]. Approximately, one-third of MDD patients exhibit resistance to conventional antidepressants, which are further limited by significant side effects and a delayed therapeutic onset, reducing their clinical effectiveness [9]. Notably, MDD displays pronounced sex differences in prevalence, symptomatology, and treatment response—women are nearly twice as likely to be diagnosed and often present with differing clinical profiles compared to men [10].

The global consensus indicates that MDD arises from a complex and multifactorial etiology; however, several hypotheses have been historically developed in order to explain its cause. One of them focuses on **genetic susceptibility**, suggesting that certain genetic factors may predispose individuals to depression [11]. Another well-established theory is the **monoamine hypothesis** [7], where imbalance in the monoaminergic neurotransmitters’ serotonin, noradrenaline, and/or dopamine accounts for depressive symptoms [12]. More recently, other neurotransmitters have been related with MDD as it suggests the hypothesis of **glutamatergic and cholinergic hyperexcitability alongside GABAergic dysfunction**, where it is posited that an imbalance in these neurotransmitters leads to the impaired synaptic plasticity associated with depressive symptoms [13, 14]. Moreover, the **hypothalamic-pituitary-adrenal (HPA) axis hyperactivation hypothesis** proposes that excessive stress hormone activity contributes to MDD [3]. Beyond neurotransmitters and hormonal factors, the **inflammation hypothesis** suggests that an overactive immune system and elevated levels of pro-inflammatory cytokines might be involved in MDD development [15]. Finally, the **circadian hypothesis** links altered circadian rhythms to the onset and progression of depression [16]. Regarding the complexity of MDD, it is likely that different subtypes of MDD with distinct biochemical profiles exist [17]. Such heterogeneity may explain the substantial variability in patients’ responses to antidepressant treatments [18].

In this complex scenario, studies involving animal models of MDD are highly valuable to gain better knowledge of this neuropsychiatric disorder. However, it is important to acknowledge that some MDD symptoms, such as guilt, suicidality, and sadness, are likely uniquely human traits [19]; nevertheless, other core features of MDD, including anhedonia, hopelessness, helplessness, as well as sleep, memory, social and appetite disturbances, can be successfully reproduced in rodents, making them a suitable model for testing refined and novel antidepressant treatments [19]. Currently, several validated protocols are available for studying MDD in rodents, which can be classified into three major categories: acute stress-based models (forced swimming test, tail suspension test, learned helplessness model), chronic stress models (chronic mild stress (CMS) paradigm, psychosocial stress models), and secondary or iatrogenic depression models (dysregulation of the hypothalamic-pituitary-adrenal axis, chronic glucocorticoid administration, retinoic acid derivative administration, immune system alterations) [19, 20].

MDD has been traditionally examined as a neuropsychiatric disease, and as such neurons were the focal point of research. However, evidence collected over the last years has highlighted the relevance of glial cells, and in particular astrocytes, in both the pathophysiology and pathogenesis of MDD [21, 22]. Astrocytes participate in a wide variety of functions in the central nervous system (CNS), from transmitter uptake, ionic buffering, metabolic support, neurotrophic factor production and modulation of synaptic transmission and plasticity to neuronal circuitry and animal behaviour regulation [23].

Remarkably, astrocytes express receptors for glucocorticoids making them direct targets to the stress hormones, corticosterone and cortisol, and consequently highly sensitive to brain stressors [24]. Thus, our understanding of MDD is undergoing a significant shift, from a predominantly neurocentric view to a holistic one that also recognizes the critical role of astrocytes [22] and other glial cells [25]. In this review, we will summarize the current view of astrocytes in the pathophysiology of MDD and their potential as therapeutic targets.

## Astrocytes in Depression

**Fig. 1:**
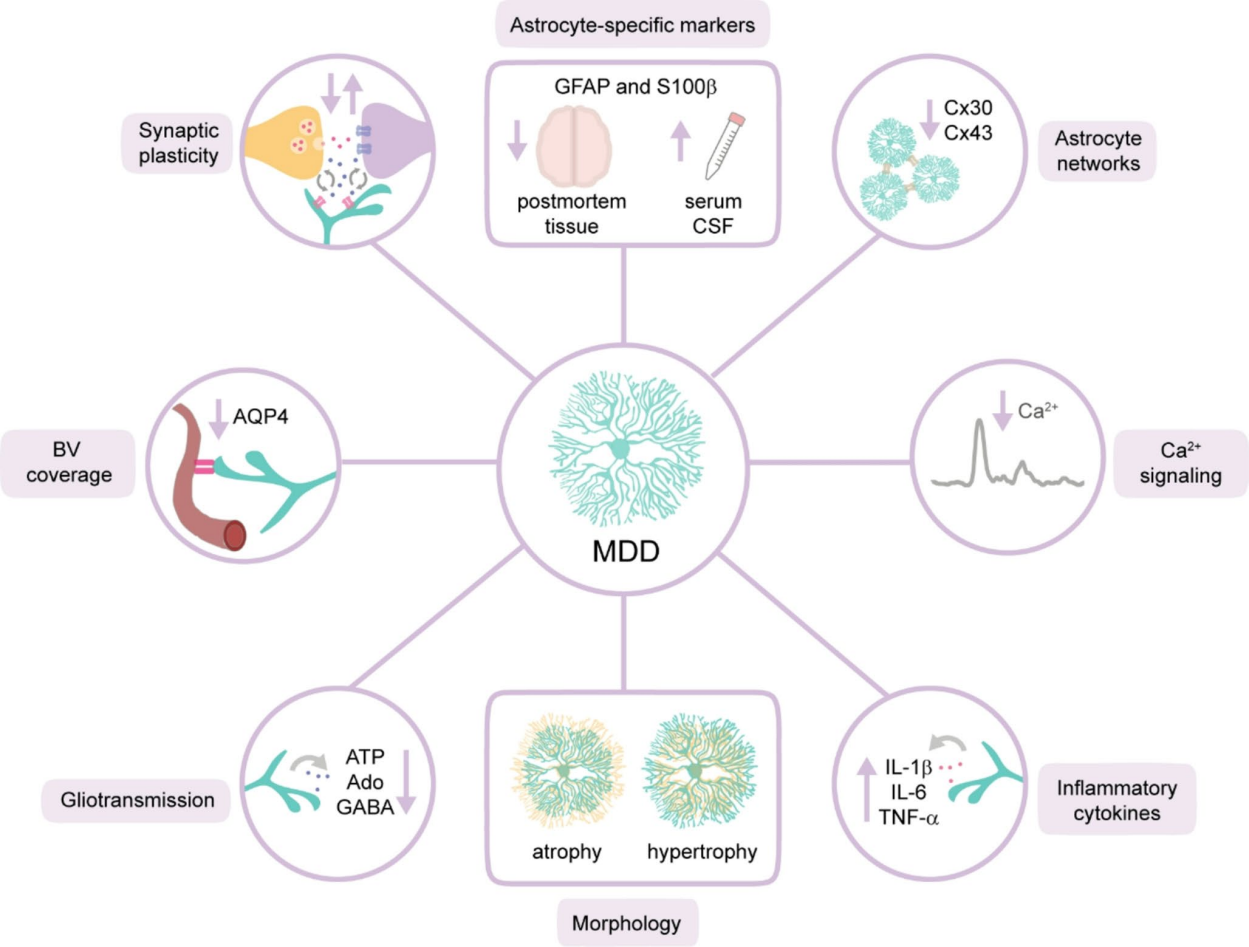
Astrocytic dysfunctions in MDD. In patients with MDD, astrocytes show altered cellular density, indicated by changes in the astrocyte-specific markers GFAP and S100β—manifesting as reduced packing density in postmortem brain tissue, but elevated levels in CSF and serum. Morphological abnormalities are also present in astrocytes, exhibiting either atrophy or hypertrophy in affected brain regions. Beyond structural changes, astrocyte functionality is disrupted in MDD, including impaired astrocytic networks, dysregulated Ca²⁺ signaling, increased release of pro-inflammatory cytokines, abnormal gliotransmission, and compromised synaptic plasticity. Abbreviations: Ado: adenosine, AQP4: aquaporin 4, ATP: adenosine triphosphate, CSF: cerebrospinal fluid, Cx30 or Cx43: Connexin 30 or 43, GABA: gamma-aminobutyric acid, GFAP: glial fibrillary acidic protein, Interleukin-1 beta: IL-1β, Interleukin-6: IL-6, Tumor necrosis factor alpha: TNF-α

Several lines of evidence have found astrocytic dysfunctions in MDD, from alterations in astrocyte density and morphology to glial function (Fig. 1).

### Alterations in Cellular Density and Morphology

Postmortem data from *MDD patients* and *suicide victims* show changes in density and morphology of astrocytes from different brain regions, such as prefrontal cortex (PFC), anterior cingulate cortex (ACC), hippocampus and amygdala [1, 26–28]. Accordingly, the expression of astrocyte-specific markers, such as glial fibrillary acidic protein (GFAP) and Ca^2+^-binding protein, S100β, has been found altered in this mental illness. Indeed, MDD human tissue have revealed a **reduced packing density of GFAP-positive cells and lower GFAP protein levels** in the PFC and CA1 and CA2 subregions of the hippocampus [28–30]. Furthermore, a 5-fold downregulated GFAP- immunoreactivity (IR) was found in the mediodorsal thalamus and caudate nucleus of suicide completers [22, 31], as well as in the cerebellum, where GFAP levels were also reduced by 32% in depressed versus control subjects [32]. Interestingly, depressed subjects under 60 years old showed higher magnitudes of GFAP reduction in PFC compared to age-matched controls [27, 29]. In contrast, older patients with a late-onset depression showed increased GFAP-IR and cell density in dorsolateral PFC [27, 33], pointing out the importance of age in MDD onset for its prognosis. However, **increased GFAP** levels have been detected in the **cerebrospinal fluid and serum** of MDD patients [34, 35] potentially due to astrocytic degeneration or to active regulation [35].

*Depressive-like animal models*, such as chronic variable stress (CVS) paradigm, chronic unpredictable stress (CUS) or early maternal deprivation mirror these findings, showing decreased GFAP expression and GFAP-density in limbic and cortical areas [36–38]. Depressed-like state rats showed lower astrocyte counts and GFAP-IR in the hippocampus, medial PFC, and cerebellum compared to controls​ [39]. Interestingly, when these rats had access to voluntary running exercise showed not only a recovery of astrocyte number and GFAP levels in all these regions, but also a relief of main depressive symptoms, suggesting that astrocyte changes are reversible and highly tied to depressive behaviour​ [39].

Damaged astrocytes leak **S100β** into the extracellular space and cerebrospinal fluid, reaching the bloodstream [40]. In accordance, *MDD patients* show elevated S100β levels in cerebrospinal fluid and serum [40, 41]. Excessive extracellular S100β results detrimental for surrounding cells, leading to neuronal apoptosis, increased production of the pro-inflammatory prostaglandins [41], and reduced integrity of the brain blood barrier (BBB), increasing the permeability to circulating toxins or peripheral inflammatory signals into the brain [42, 43]. On the other hand, postmortem studies showed a reduced transcription of S100β in the PFC of MDD patients [44, 45] and reduced S100β + cell density in the CA1 pyramidal layer [46].

In *depressive-like rodent models* such as olfactory bulbectomy (OBX) or CUS in rats, increases in serum S100β levels have been observed [47], mirroring findings in depressed patients. However, based on the model studied, significant but contradictory changes in S100β can be found. Indeed, the chronic unpredictable mild stress (CUMS) rat model shows reduced expression in the dentate gyrus but, following stress re-exposure—mimicking recurrent depression—a rebound increase in S100β + astrocytes was observed [48]. In this line, maternal separation stress protocol in rats deplete S100β expression in mPFC astrocytes [49], further highlighting the impact of chronic stress on astrocyte physiology.

Alteration in astrocyte density is generally accompanied by changes in **astrocyte morphology**. There are discrepancies in this aspect, depending on the area (mPFC, hippocampus, amygdala) and the origin of the analysed tissue, i.e., depressive patients or animal models of depression. While some studies report astrocytic atrophy based on GFAP morphological reconstruction with reduced soma size and astrocyte processes [50–53], others show astrocyte hypertrophy based on an increase in the soma and the number and length of processes [26, 54, 55]. Astrocyte morphology changes associated with MDD may in turn have an impact on astrocyte functionality. It is known that distribution of astrocytes in the CNS is highly organised. Protoplasmic astrocytes occupy separate three-dimensional non-overlapping territories that define functional (anatomical) domains [56], where the somas and main processes do not come into contact with each other and only the most peripheral processes intersect. Endo et al. (2022) identify astrocyte territory size-related genes and how some of these genes can be affected in different diseases, including MDD, indicating that astrocyte morphological changes may represent a common feature of different CNS disorders [57]. Furthermore, astrocyte hypertrophy leads to newly proliferated astrocytes, which might disrupt astrocyte functional domains causing long-lasting reorganization of tissue architecture [58]. On the other hand, atrophy impacts the perisynaptic astrocyte processes (PAPs) geometry, which can reduce synaptic and neuronal coverage, altering glutamate and K + homeostasis, ultimately affecting synaptic transmission and neuronal activity [53, 59]. In CUS mouse model, astrocytes show a reduced expression of ezrin, a plasmalemmal-cytoskeleton linker that assembles and stabilises membrane-cytoskeletal complexes [59]. Notably, modifying ezrin expression in astrocytes from PFC has an impact in how mice respond to chronic stress. Thus, increased expression levels of ezrin protect animals from developing depressive-like behaviours, while its reduction renders them susceptible to stress [60], highlighting the critical role of astrocyte geometry for stress related disorders.

Besides the benefits of using GFAP as astrocyte marker to several studies, this approach also shows significant limitations. GFAP expression does not correlate with the actual number of astrocytes, nor does it fully capture their complex morphology, particularly at the fine processes [61]. Therefore, an important effort has been made to apply new advanced tools for a comprehensive 3D reconstruction of astrocyte morphology with high spatial resolution, including genetically encoded reporters, intracellular dyes microinjection, or viral labelling with fluorescent proteins [53, 62–65]. These approaches have discovered undisclosed features of depression-associated changes in astrocytes, like reduced astrocytic territorial domains, astrocyte arborisation complexity and the loss of perisynaptic leaflets in mice subjected to chronic stress [53, 60, 63].

### Dysregulated Glutamate and GABA Homeostasis

Neuronal signalling is highly regulated by astrocytes. Through the glutamate/GABA-glutamine cycle, astrocytes can take up synaptically released glutamate and γ-aminobutyric acid (GABA) and metabolize them into glutamine by the glutamine synthetase (GS). Glutamine is taken back by neurons and converted into glutamate or GABA by the neuron-specific phosphate-activated glutaminase [66]. This exchange of glutamate, GABA, and glutamine between neurons and astrocytes is crucial for maintaining excitatory and inhibitory neurotransmission, but also to avoid excitatory overstimulation and concurrent excitotoxic damage [66, 67]. Astrocytes are enriched in glutamate transporter GLT-1 and glutamate-aspartate transporter GLAST that contribute to the fast uptake of glutamate from the synaptic cleft [66]. These astrocyte-specific glutamate transporters correspond to the excitatory amino acid transporters EAAT2 and EAAT1, respectively, in the human brain [68].

Postmortem studies in *MDD patients* have found a decrease in several components of the glutamate/GABA-glutamine cycle, such as EAAT2, EAAT1 and GS in the frontolimbic region [69, 70]. These results are corroborated by neuroimaging studies in MDD patients that show decreased glutamate and glutamine levels in different regions, such as the PFC and hippocampus [71, 72]. Microarrays studies of postmortem tissue from MDD patients have also found a significant downregulation of a subset of genes encoding the glial high-affinity glutamate transporters SLC1A2 (EAAT2; GLT1), SLC1A3 (EAAT1; GLAST) and GS [69]. Additionally, a dysregulation of genes encoding various subunits of glutamate receptors and GABA_A_ receptors has been described [69]. Consistent with these results, some studies have shown that inhibition of GLT-1 and/or GLAST transporters in astrocytes in specific regions of the PFC induces depressive states in *animal models*, manifested by increased hopelessness and anhedonia [73, 74]. Moreover, chronic exposure to corticosteroid or dexamethasone (a synthetic glucocorticoid) or the chronic learned helplessness rat model of depression lead to a significant reduction in the levels of GLT-1 [75] and GLAST proteins, as well as their mRNAs in PFC and hippocampus [76, 77]. Overall, the reduced glutamate uptake results in an excess of glutamate in the extrasynaptic space, leading to an overactivation of extrasynaptic receptors and neuronal excitotoxicity [74, 78].

Alterations of the GABAergic system can also contribute to the pathophysiology of MDD [79]. Indeed, imaging studies of *MDD patients* have confirmed reduced GABA levels in several cortical regions [80, 81]. Furthermore, the GABA_A_ receptor subunits GABA_A_β3, GABA_A_δ, and GABA_A_γ2 were upregulated in dorsolateral PFC in postmortem tissue, while the GABA_A_α1 and GABA_A_β3 subunits were upregulated in ACC from suicidal subjects diagnosed with MDD [69].

Dysfunction of GABAergic signalling has also been confirmed in *rodent models of depression.* Animals exposed to CMS exhibited lower hippocampal GABA levels compared to controls [79, 82], and decreased cortical GABA_A_ receptor function, reduced release probability at peri-somatic GABAergic synapses, and diminished postsynaptic GABA_B_ receptor mediated inhibition, leading to an increased excitability of pyramidal neurons [83]. Similarly, early-life stress, including maternal separation stress and altered maternal care during the first postnatal weeks, led to a decreased GABA_A_ receptor expression in the frontal cortex and other limbic areas, as well as depressive-like behaviours and higher levels of anxiety in adulthood [84, 85]. Notably, rats that went through CUS or CMS or with congenital helpless behaviour (cH), a genetic rat model for human depression, displayed significantly lower expression of the GABA transporter GAT3 in PFC, compared to control littermates, which may be associated with reduced GABA levels in mice with depression-like behaviours [86–88].

Astrocytes are critical for regulating GABA homeostasis, as they can uptake, synthesize and release GABA, contributing to the final outcome of GABAergic signalling [89]. In the Flinders Sensitive Line (FSL) rat model of depression, reactive astrocytes exhibit increased activity of the monoamine oxidase-B (MAO-B) enzyme, leading to an excessive synthesis and release of GABA. This, in turn, enhances tonic inhibition through extrasynaptic GABA_A_R activation [90]. Interestingly, either blocking astrocytic intracellular Ca^2+^ signalling or reducing astrocytic GABAergic activity through MAO-B enzyme inhibition, tonic inhibition can be reduced, and the impaired synaptic plasticity could be restored in the FSL PFC [90]. However, it remains unknown whether this restoration of synaptic plasticity through MAO-B inhibitors might also recover the main symptoms associated with depression.

### Hemichannels and water Homeostasis Imbalance

An important feature of astrocytes is their ability to establish large functional networks through gap junctions [91]. Such connectivity allows astrocytes to fine tune potassium, calcium and water homeostasis, as well as metabolite transport [1, 92] across hundreds of microns. In MDD, the ability of astrocytes to communicate intercellularly via gap junctions is altered, which might contribute to the pathophysiology of this illness [93]. Expression levels of Cx43 and Cx30 proteins, the two predominant gap junctions subunits in astrocytes, as well as their mRNAs have been found reduced in postmortem tissue from *MDD patients* and *animal models of depression* [94, 95] in brain regions involved in mood regulation, such as the PFC [93], orbitofrontal cortex [96], hippocampus [94], locus coeruleus [97], mediodorsal thalamus and caudate nucleus [98]. In addition, the reduced expression of connexins in MDD may cause a disruption from the molecular level to behaviour. Supporting this, several rodent studies showed that CUS, CSDS, chronic restraint stress protocol or chronic corticosterone treatment induce a significant decrease in Cx43 protein, accompanied by gap junction dysfunction in the PFC and hippocampus [93, 95, 99]. Furthermore, in CSDS mice, the reduced expression of astrocytic Cx30 and Cx43 causes depression of neuronal activity in mPFC and hippocampus [95]. Interestingly, the overexpression of these connexins not only increases local neuronal activity in both brain areas but also reverses depressive-like behaviours in CSDS mice. To further support these data, the downregulation of astrocytic Cx30 and Cx43 in PFC and hippocampus of healthy mice was able to induce depressive behaviours [95], which can be similarly observed by the infusion of non-selective gap junction coupling inhibitors into the PFC of healthy rats [93].

**Water homeostasis**, which is tightly regulated by aquaporins, is closely intertwined with astrocytic gap junctions contributing to intercellular water flows. Aquaporin-4 (AQP4) is a plasma membrane water regulating channel, specifically localized and enriched in the endfeet of astrocyte processes [100]. This strategic localization enables AQP4 to regulate water and electrolyte homeostasis, the extension and migration of astrocyte processes during neuronal activity, glutamatergic transmission and BBB integrity [101]. In the context of depression, preclinical and clinical studies have suggested a dysfunctional activity of AQP4 [102]. In *MDD patients*, postmortem analyses of brain tissue have revealed reduced coverage of blood vessels by AQP4-positive astrocytic endfeet in the PFC grey matter [45]. Additionally, decreased AQP4 protein and gene expression have also been observed in the hippocampus [94] and locus coeruleus [97].

Similarly, in *depressive-like animal models*, CMS leads to a decreased AQP4 protein expression in several brain areas, including PFC, locus coeruleus, choroid plexus and hippocampus [102–105]. Rats selectively bred for high anxiety-like behaviour (HAB) showed a reduced AQP4-IR astrocyte coverage of blood vessels in the adult PFC compared to controls [101]. Interestingly, AQP4 KO mice, which show important astrocytic dysfunctions [106, 107], present cognitive deficits similar to those observed in mood disorders [107], and display an exacerbated depressive-like behavioural response after corticosterone treatment. Furthermore, an immune-related model of depression by intracerebroventricular injection of the tumor necrosis factor (TNF)-like weak inducer of apoptosis (TWEAK) significantly decreased AQP4 protein expression and increased anhedonic behaviour [108].

Regarding synaptic plasticity, reduced AQP4 expression impairs astrocyte ability to regulate and support synaptic function. AQP4 KO mice exposed to chronic high levels of corticosterone shown downregulated excitatory amino acid transporter 2 (EAAT2) in the hippocampus​ [106]. This impaired synaptic glutamate clearance may lead to glutamate spillover and excitotoxic or maladaptive signalling. Interestingly, AQP4 KO mice show slower rates of extracellular K⁺ removal during neural activity, leading to higher extracellular K⁺ after synaptic stimulation and favouring neuronal hyperexcitability​ [109]. Additionally, absence of AQP4 drives reduced levels of synapsin-1 and glial-derived neurotrophic factor (GDNF), as well as decreased hippocampal neurogenesis, indicating that AQP4 deficiency compromises both synaptic plasticity and adult neurogenesis [106], key processes for mood regulation. Consistently, earlier studies found deficits in long-term potentiation (LTP) of synaptic plasticity and altered neurotrophin signalling in AQP4 KO mice, which correlated with impairments in learning and memory processes [107, 110].

AQP4 signalling is highly sensitive to neuroinflammation. Indeed, astrocytes lacking AQP4 showed attenuated cytokine response in vitro, which reduced the release of tumor necrosis factor-α (TNFα) and interleukin-6 following lipopolysaccharide (LPS) challenge compared to wild-type astrocytes [111]. In this line, CUS in rodents increases neuroinflammatory markers while reducing AQP4 polarization in astrocytes, thereby promoting oxidative stress and inflammation [112]. Finally, a dysfunctional AQP4 signalling also impaired glymphatic clearance, which in chronic stress can lead to the accumulation of inflammatory mediators and oxidative stress processes in the brain [112]. Although the underlying mechanisms are not fully understood, these findings highlight the direct relationship between AQP4 activity and depressive behaviour, pointing out astrocytic AQP4 as a potential mechanistic target for the pathophysiology of depression.

Astrocytes are critical components of the neurovascular unit that support **BBB** function. It is known that BBB integrity is compromised in MDD [42]. Indeed, magnetic resonance imaging in *MDD patients* has shown abnormal permeability of the BBB and how vascular leakage can be associated with depression and its severity [113]. Additionally, altered BBB function leads to elevated levels of circulating biomarkers, as S100β, albumin and proinflammatory levels in blood and CSF [42]. Interestingly, transcriptomic profiling of postmortem female brain samples revealed altered endothelium gene expression, suggesting sex-specific BBB vulnerability associated to MDD [114]. Similarly, in female *rodents*, chronic social-defeat stress (CSDS) can induce loss of Claudin-5 (Cldn5), the dominant tight junction in BBB, in the PFC and NAc leading to BBB leakiness in these brain areas and promoting anxiety and depressive-like behaviors [114].

### Dysfunctional Energy Metabolism

Astrocytes and neurons maintain a reciprocal metabolic relationship, with astrocytes playing a key role in energy supply and synaptic function. When uptaking glucose from capillaries, astrocytes can either release it, metabolize it through glycolysis to generate adenosine triphosphate (ATP), or convert it into lactate, the latter being an essential neuronal energy substrate that also modulates synaptic transmission and plasticity [115]. Through imaging studies in *MDD patients*, impaired glucose metabolism has been observed in the PFC, amygdala and hippocampus [116–118], a reduced astrocytic endfeet coverage of blood vessels [45], and decreased levels of ATP-derived nucleoside triphosphate [119]. Also, recent research has identified impairments in mitochondrial function, including reduced respiration and decreased oxygen consumption linked to ATP production in MDD patients [120]. Notably, enhancing brain energy metabolism has been associated with an improvement in symptoms of MDD patients [121], underscoring the importance of astrocytic metabolic support in maintaining proper neuronal function and mood regulation.

Likewise, chronic stress in *animal models* has been shown to downregulate the expression of connexins Cx43 and Cx30, disrupting lactate transport from astrocytes to neurons and impairing synaptic plasticity [1, 54]. Furthermore, chronic social-defeat stress (CSDS) causes reduced levels of glycolysis and lactate dehydrogenase A (LDHA) expression — a glycolytic enzyme that catalyses L-lactate production — in the dorsomedial PFC [122]. LDHA deficiency in astrocytes reduces L-lactate production leading to impaired neuronal excitability through elevation of BK channel-mediated fast after-hyperpolarization (fAHP) and eventually contributing to the development of depression-like phenotypes [122]. Supporting this relationship, exogenous lactate infusion into the dorsomedial PFC significantly alleviates the depression phenotype in mice [122].

### Dysregulated Neuroinflammatory Pathways

Supporting the inflammatory hypothesis in MDD [15], some studies have reported elevated pro-inflammatory cytokines, such as IL-1β, IL-6 and TNF-α and inflammatory markers like c-reactive proteins, in *MDD patients* [123]. In line with this theory, astrocytes, based on their ability to produce and release cytokines and their close interaction with microglia, have been considered as important players in MDD [124]. The isolated extracellular vesicles derived from astrocytes from MDD patient serum show increased levels of different inflammatory markers (Interferon-γ, IL-1β, IL-2, IL-6 and TNFα) [125]. Moreover, a human genetic variant in the astrocyte-expressed multiple endocrine neoplasia type 1 (men1; protein: menin) gene has been linked to depression risk [126]. This variant disrupts the tumour suppressor protein menin inhibition of nuclear factor-κB (NF-κB), which is a critical regulator of inflammation in CNS [126], potentially enhancing NF-κB activation and interleukin-1β production in astrocytes​ [126]. These findings align with data from *CUMS or endotoxin (LPS) exposure in mice* showing a reduced astrocytic menin protein levels, also accompanied by over-activation of NF-κB and excessive release of IL-1β from astrocytes [126]. Remarkably, by treating those mice with an NF-κB inhibitor or an IL-1β receptor antagonist it was possible to reverse the depressive phenotype ​ [126]. Another study found that LPS exposure in mice promoted the transition of astrocytes into a neurotoxic and reactive state, characterized by reduced brain-derived neurotrophic factor (BDNF) expression and elevated IL-1β and TNF-*α* levels [127]. Thus, inhibition of activated astrocytes significantly attenuated neuroinflammation and GFAP levels, whereas maintained BDNF expression and ameliorated LPS-induced depressive-like behaviours [127]. Additionally, in the CUMS model, astrocytes excessively secrete complement C3, which binds to C3a receptors on microglia promoting a pro-inflammatory state [128]. Nevertheless, infusion of human umbilical cord mesenchymal stem cells (hUC-MSCs), which release anti-inflammatory factors, interrupted the C3–C3aR interaction resulting in microglial activation towards an anti-inflammatory state, alleviating neuronal damage, synaptic deficits and depressive behaviour [128]. Altogether, these data reveal astrocytes as attractive therapeutic targets to modulate inflammatory processes in MDD.

### Impaired Intracellular Ca^2+^ Signalling

Intracellular Ca^2+^ has been revealed as a common signalling pathway for astrocytes to dynamically interact with the microenvironment [129], with important consequences for neuronal circuits and behaviour [130]. Extensive knowledge of astrocytic Ca²⁺ dynamics in the context of neurodegenerative and neurodevelopmental disorders, like Alzheimer’s disease, epilepsy, autism spectrum disorder, or Huntington’s disease, can be found in the literature [131–134]. On the contrary, Ca^2+^ signalling and its characteristics in MDD remain less explored even though in the last few years, emerging evidence points to a disrupted astrocyte Ca²⁺ signalling as a major contributor to depressive-like behaviours [135–137]. For instance, it has been demonstrated that corticosterone-treated mice (Cort-mice) exhibit significantly reduced social-driven astrocytic Ca²⁺ activity in mPFC [135]. In contrast, astrocyte Ca²⁺ dynamics remained unaffected during object interactions, showing a direct link between the value of interactions and astrocyte Ca^2+^ signalling during natural behaviour [135]. Similarly, in CSDS model a decreased astrocytic Ca²⁺ signalling in the dorsal raphe nuclei (DRN) both during exploratory behaviour in the open field test and during social interaction has been found [138]. These findings suggest that Ca²⁺ hypoactivity in astrocytes is a consistent feature across different models of depression. Indeed, restoring astrocyte Ca²⁺ signalling through selective chemogenetic activation can reverse the behavioural deficits of these mice. Thus, boosting the Gq-mediated Ca²⁺ activity of mPFC or DRN astrocytes improves social interaction, reverse anxiety and anhedonia levels in both Cort and CSDS mouse models [135, 138].

Astrocytic Ca^2+^ signalling also plays a pivotal role during brain development. Luo et al. (2023) showed that dampening cortical astrocytic Ca²⁺ activity during early development via overexpression of plasma membrane calcium-transporting ATPase 2 (PMCA2) led to depressive-like behaviours, impaired social interaction, and synaptic abnormalities in adulthood. However, chemogenetic activation of astrocytic Ca²⁺ signalling rescued synaptic and behavioural deficits in these mice, underscoring its importance for neural circuit development and its potential link to the pathogenesis of neuropsychiatric disorders [136]. In this line, mice lacking IP3 receptor type-2 (*Itpr2*^−/−^ mice), which show a profound downregulation of astrocyte Ca^2+^ signalling, display depressive-like phenotypes accompanied by abnormal brain-wide functional connectivity patterns and reduced resting-state connectivity in frontal-limbic circuits, both reminiscent of alterations observed in MDD patients​ [139].

Additionally, systemic inflammation by peripheral LPS exposure triggers astrocyte reactivity through Orai1 calcium channels [137]. Interestingly, astrocyte-specific Orai1 KO mice were resilient to LPS-induced depressive-like behaviours and diminished expression of proinflammatory cytokines. These findings suggest that astrocyte Ca^2+^ channels are critical to understand the link between neuroinflammatory responses and mood regulation. Overall, these findings highlight that astrocyte Ca²⁺ signalling is not only a biomarker of MDD, but also a potential therapeutic target to ameliorate this illness.

### Disrupted Tripartite Synapses

The concept of tripartite synapse, which describes the bidirectional exchange of signals between astrocytes and neurons [140], challenged the neurocentric view of neurons as the unique mediators of information transmission in the nervous system. Nowadays, the role of astrocytes has been accepted and extended to the entire nervous system, being critical in regulating different behaviours [141]. The tripartite synapse framework has been expanded including microglia [142] and oligodendrocyte precursor cells (OPCs) [143], based on their active participation in synaptic function, leading to a broader concept, the multipartite synapse [144].

Astrocytes can sense neuronal activity and, in addition, modulate neuronal excitability and synaptic plasticity by releasing diverse neuroactive substances, so-called gliotransmission [145, 146]. To orchestrate this, astrocytes express membrane receptors and transporters for a wide range of neurotransmitters and neuromodulators, such as glutamate, GABA, adenosine (Ado), noradrenaline (NA), serotonin (5-HT), acetylcholine (ACh), or endocannabinoids (eCBs) [135, 147]. Most of those are linked to the release of Ca²⁺ from astrocytic intracellular stores, which results in autocrine or paracrine actions, including neurons and other glial cells. Particular attention has been paid to the purinergic and glutamatergic astrocyte signalling in the context of MDD [52, 148]. Imaging studies have reported a positive correlation between depleted ATP levels in frontal cortical brain regions and *MDD in adult patients* [119, 149]. Samples from suicide subjects show significant lower expression of *ENTPD2* gene, primarily expressed in astrocytes within the deep gray matter [150]. *ENTPD2* hydrolyses ATP to ADP and AMP, thereby regulating purinergic signalling by controlling nucleotide availability and as such reduced *ENTPD* levels may affect the amount of ATP available impairing purinergic receptor activation [150].

Likewise, diverse evidence from *rodent models of MDD* corroborates on the close relationship between astrocyte purinergic signalling and depression. Thus, CSDS mice exhibited reduced ATP levels, particularly in the PFC and hippocampus brain slices [21, 134]. Boosting astrocyte-promoted purinergic activity in *Itpr2*^−/−^ mice was sufficient to induce antidepressant effects via P2 × 2 receptors [21]. Furthermore, deficiencies in ATP release by astrocytes in animal models with MDD-related behaviours, like *Itpr2*^−/−^ mice or transgenic mice with a blockade of vesicular gliotransmission (dn-SNARE mice), can be reversed by i.p. ATP administration [21]. On the other hand, lack of insulin receptors in astrocytes causes increased anxiety and depressive-like phenotype behaviour, due to decreased purinergic signalling from astrocytes to dopaminergic neurons [151], and in mPFC, an impaired epoxyeicosatrienoic acid (EET) signalling affect ATP release from astrocytes leading to depressive-like behaviour [152]. Moreover, absence of glucocorticoid receptors in astrocytes has been associated with depressive-like behaviours, that occurred due to the reduced ATP release driven by downregulated astrocytic Ca^2+^ activity in response to stress [24]. Concomitantly, ATP can be released by non-exocytotic pathways, which have been also found altered in depression. Thus, in the CUS model the expression of Cx43 and Panx-1 channels with high permeability to ATP was reduced in PFC [96, 153], and by blocking Cx43 and Panx-1 channels in control animals depressive-like behaviour can be induced [154, 155]. Although the underlying processes remain not fully understood, these studies reveal the importance of astrocytic ATP homeostasis for the pathophysiology of MDD.

Another key actor that could participate in modulating depressive-like behaviours is adenosine, as part of the purinergic signalling, which can be released by both astrocytes and neurons [156–158]. Remarkably, elevation of astrocyte-derived adenosine levels and upregulation of adenosine receptor A1 signalling have been revealed as effective antidepressive treatments in animal models [159, 160]. In addition, non-pharmacological antidepressant treatments for MDD, such as sleep deprivation or deep brain stimulation of the infralimbic PFC, depend on an unaltered neuronal-glia signalling. In particular, astrocytes modulate deep brain stimulation effects involving adenosine A1 receptors [160], and administration of A1 receptor agonist can mimic the antidepressant effect of sleep deprivation [159–161].

Dysregulation of glutamate and serine release by astrocytes can have also an impact in mood disorders [69, 162]. Indeed, mice treated with corticosterone show an enhanced release of glutamate/D-serine compared to control mice [135]. Additionally, in the FSL rat model, GLAST down-regulation contributes to astrocytic glutamate dysregulation by reducing glutamate reuptake, contributing to the increased glutamatergic synaptic activity in CA1 hippocampal area [163]. By knockdown of astroglial GLT-1 and GLAST transporters in vivo it is possible to both increase gliotransmission and the excitatory neuronal transmission in mouse infralimbic cortex, mimicking MDD phenotype [74]. Additionally, in chronic restrain stress model, increased astrocytic Cx43 hemichannel activity in the ventral hippocampus leads to an excessive astrocytic glutamate and D-/L-serine release [164]. This excess of extracellular glutamate can overactivate postsynaptic NMDA receptors and trigger depressive-like symptoms. Notably, the pharmacologically blockage of astrocytic Cx43 hemichannels during chronic restrain stress protocol prevented the development of depressive behaviours and normalized extracellular glutamate levels​ [164]. Altogether, these findings suggest that an exacerbated astrocytic glutamate release might contribute to the aberrant synaptic excitation found in depression [163, 164], supporting the use of NMDA antagonists, i.e., ketamine, as effective antidepressant treatments [165].

## Concluding Remarks

MDD is a complex and heterogeneous disorder, which makes it difficult to fully understand or treat through a single point of view and a single cellular player. This review highlights the growing evidence pointing to astrocytic dysfunction as a key factor in the pathophysiology of MDD. Studies in both postmortem human brain tissue and rodent models consistently report alterations in astrocyte density, morphology, and expression of glial markers. These findings support the potential utility of astrocyte-specific markers as diagnostic or prognostic biomarkers for depression.

However, it is important to consider different factors that may affect these astrocytic biomarkers. First, the analysis of postmortem tissue from MDD patients, being highly valuable, it shows critical limitations, such as the cause of *death*, which can be different (i.e., suicide victims) [22, 26, 150, 166], and the interval occurred until the brain tissue is processed, affecting the quality of RNAs obtained [167]. Another important fact is the *age* of the patients and the onset of depression, that can affect the reduction of packing density of GFAP expression [27, 29]. Moreover, when interpreting or generalizing findings from postmortem brain tissue other confounding factors have to be considered, like the antidepressant treatment (doses and period) [168], family history of mental disorders [8], environmental factors, or alcohol and other drugs of abuse [29]. Last but not least, the *sex* is a key differential factor that can account for multiple changes between individuals [169]. Epidemiological and meta-analysis data suggest that the incidence of MDD in women was approximately twice as high as that in men [170, 171]. Such difference is related to genetic factors, sex hormones, and heightened exposure to severe adversity [172]. Also, clinical manifestations of depression differ by sex, being women more likely to report anxiety symptoms, guilt, atypical depression, and somatic symptoms, while men are more prone to exhibit suicidal and addictive behaviours [173, 174]. These differences can hinder the accurate diagnosis and treatment of MDD [173–175]. Moreover, women and men respond differently to antidepressant treatment [10]. In particular, women tend to respond better to selective serotonin reuptake inhibitors (SSRI) and MAO inhibitors, being more responsive to fast acting antidepressants such as ketamine. In contrast, men usually respond better to tricyclic antidepressants (TCA) [176]. In order to improve diagnostic precision and develop individualized treatment for men and women more studies are now focusing on sex-specific differences. Thus, sex stratified genome-wide association studies (GWAS) have identified 11 loci associated with depression in women compared to just one in men [177], and sexually dimorphic transcriptional profiles have been found for genes involving synapse related pathways and immune-related pathways [178], which might open new venues for sex-specific treatments for MDD.

Regarding astrocytes, sex-differences in S100β serum levels from MDD patients have been reported [179]. Additionally, astrocyte-specific genes were upregulated in men but remained unchanged in women from brain samples of MDD patients [180]. In this context, alterations in BBB found in MDD show marked sex differences regarding soluble biomarkers and transcriptomic profiles both in humans and mice [114]. All these data highlight the importance of including sex as a biological variable in MDD research, being crucial for advancing precision medicine with unbiased health outcomes.

Caution should also be taken when studying the animal models, where the age of animals at the onset of the treatments, the sex as well as the duration of the treatments or approaches to induce depression (olfactory bulbectomy, maternal separation, learned helplessness, repeated restraint stress, chronic unpredictable stress, social isolation, chronic social defeat stress, witness defeat, genetic and pharmacological rodent models) can have an impact in astrocyte pathophysiology, accounting for the diverse and sometimes contradictory results [181]. Most of the animal models of depression have been developed using males and later applied to their female counterparts [181]. As a result, certain models regularly used and accepted to evaluate depressive behaviours, such as forced swimming test (FST) and CMS paradigm [182], or CSDS and learned helplessness [183, 184] are less suitable for studying depression in females, as females show distinct responses and adaptations to stress, and in most strains, rodent females do not readily engage in aggressive encounters or exhibit learned helplessness in the same way than males do.

Another important aspect to disclose is the limited and often transient efficacy of current antidepressants. MDD likely involves multiple interacting systems, where targeting a single molecule or pathway is challenging. Moreover, many animal models oversimplify depression by relying on brief, stress-induced behavioural changes without capturing the disorder’s chronicity or complexity. Novel paradigms as the Depression-Like Syndrome (DLS) model proposed by von Mücke-Heim et al. (2023) offer a promising way to follow [185]. By aligning with the Diagnostic and Statistical Manual of Mental Disorders 5th version (DSM-5), the International Classification of Diseases 11th revision (ICD-11), and Research Domain Criteria (RDoC) approach, this model provides a more nuanced, multidimensional framework for studying depression-like behaviour in rodents, incorporating duration, social and functional impairment, biological validity, and symptom quantification to enhance interspecies and inter-model homology, construct, and face validity along with comparability and generalizability of findings [185].

In summary, astrocytes emerge not merely as passive bystanders of neurons but as active contributors to the development and maintenance of MDD. Future research should unravel the temporal and mechanistic relationships among different astrocytic alterations and MDD, and assess how these changes in astrocyte are impacting local networks and their functional outcome affecting behaviour. Based on this, a better understanding of the pathophysiology of depression will be possible, and more effective, and personalized treatments could be achieved.

## Data Availability

No datasets were generated or analysed during the current study.
